# Supplementary material for: MetaRibo-Seq measures translation in microbiomes
Source: Nat Commun. 2020 Jun 29;11:3268. doi: 10.1038/s41467-020-17081-z (PMC7324362; doi:10.1038/s41467-020-17081-z)
Supplement: Supplementary file 10 — Supplementary Data 7 [file 41467_2020_17081_MOESM10_ESM.zip › File2/Confidence_High_Taxonomy/123107_out.krona.html]

Javascript must be enabled to view this page.

members
magnitude
magnitudeUnassigned
count
unassigned
taxon
rank

123107\_out

3

superkingdom
3
2

phylum
1239
2

2
186801
class

2
186802
order

1
541000
family

genus
1
216851

1

SRS144297\_contig\_number\_contig-100\_5142.5143
1971605
species

species
1897045

SRS148817\_contig\_number\_contig-100\_2081.74779
1

phylum
1224
1

1236
1
class

order
135625
1

family
712
1

1
724
genus

729

SRS104039\_contig\_number\_20688
1
species
